# Modelling the impact of temperature and bird migration on the spread of West Nile virus^☆^

**DOI:** 10.1016/j.onehlt.2026.101386

**Published:** 2026-03-11

**Authors:** Pride Duve, Felix Gregor Sauer, Renke Lühken

**Affiliations:** Bernhard Nocht Institute for Tropical Medicine, Hamburg, Germany

**Keywords:** West Nile virus, Diffusion, Advection, Spread, Mosquitoes, Migratory birds, Resident birds

## Abstract

West Nile virus (WNV) is a climate-sensitive mosquito-borne arbovirus that circulates between mosquitoes of the genus *Culex* and birds, with potential spillover to humans and other mammals. Recent trends in climatic change, characterised by early and/or prolonged summer seasons, increased temperatures, and above-average rainfall, are likely to have facilitated the spread of WNV in Europe. In this work, we formulate a spatial WNV model as a system of parabolic partial differential equations (PDEs), incorporating diffusion, advection, and temperature-dependent parameters, namely the mosquito birth rate, mosquito biting rate, extrinsic incubation rate, and mortality rate. Diffusion represents the random movement of both mosquitoes and birds across space, while advection captures the directed movement of migratory birds. The model is first studied mathematically, and we show that it has non-negative, unique, and bounded solutions in time and space. Numerical simulations of the PDE model are performed using temperature data for Germany (2019–2025). The simulation results showed strong agreement with the reported WNV cases among birds and equids in Germany. The observed spread patterns throughout the years were mainly driven by the combination of temperature, diffusion processes of hosts and vectors, and the biting preference of mosquitoes between resident and migratory birds. The model better explained the observed WNV spreading pattern, including distant, isolated cases in Germany, when more bites were allocated to migratory birds than to resident birds.

## Highlights

• A PDE model is proposed to investigate the spatial spread of WNV in Germany.
• Mosquito and bird movement are modelled through diffusion processes.
• Directed migratory bird movement is incorporated through advection.
• Temperature-dependent mosquito biting, latency, and mortality are considered.
• The model predicts WNV spread in Germany and can be adapted to any region.

## Graphical abstract

**Figure:**
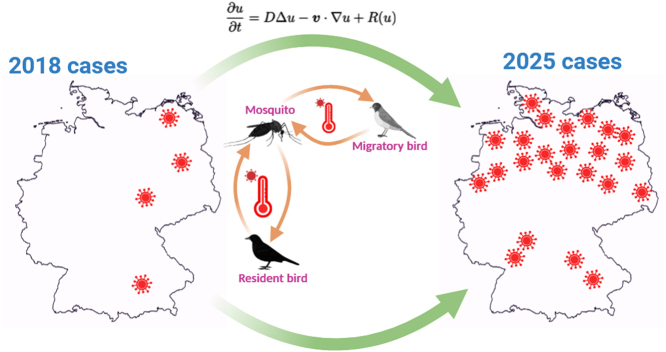

## 1 Introduction

West Nile virus (WNV) is an arbovirus of the Japanese encephalitis antigenic complex of the *Flaviviridae* family. It was first detected in a febrile patient from the West Nile district of Northern Uganda in 1937 [1], but is now considered the most geographically widespread arbovirus worldwide [2]. The virus circulates in an enzootic cycle between *Culex (Cx.)* mosquitoes and birds, with regular spillovers to humans, equids, and other mammals [3], [4], [5]. Birds are considered amplifying hosts because they develop sufficient viremia, allowing infection of mosquitoes, whereas humans, equids, and other mammals are considered dead-end hosts [6]. Most humans infected with WNV are asymptomatic, but a few may develop fever, headache, rash, among others [7], including the risk of a fatal outcome [8]. Infected equids often exhibit ataxia, lethargy, and weakness as signs of the infection [9]. Multiple theories have been proposed to explain the introduction of new WNV cases in a previously unaffected region. There is evidence that migratory birds play an important role in the spread of the pathogen [6]. For example, birds captured during their seasonal migration have tested positive for WNV in Israel [10], [11], [12], [13]. Migratory birds likely spread the virus to stopover sites in Europe as they travel from the south to the north [4], [14]. In contrast, considering the speed and duration of migratory birds’ flights, along with the duration of viremia, other researchers argued that the short-distance movements of resident birds better explain the spread pattern of WNV [6].

Several mathematical models for WNV have been formulated. Many models emphasise the role of climatic conditions in the dynamics of WNV, as temperature plays a critical role in mosquito life-history traits and virus replication. Thereby, temperature-dependent functions are used to model mosquito and virus development rates [15], [16], [17]. These are included in ordinary differential equations (ODEs) to analyse the dynamics of WNV. However, local transmission dynamics are generally modelled within the vector and host populations, without accounting for geographic spread. Fesce et al. [18] estimated the basic reproductive number of the WNV infection and used it to quantify the infection spread in mosquitoes and birds, to classify areas with temperatures suitable for the outbreak of WNV. Other studies focus on controlling WNV, e.g., Bowman et al. [19] simulated mosquito-reduction methods and personal protective measures. A potential spill-over from birds to equids and humans was modelled by Laperriere et al. [16] using temperature-driven parameters and providing values for parameters governing development and transitions between compartments. Other models analysed differences in seasonal trends using sinusoidal (trigonometric) functions to represent seasonal variations of the WNV disease dynamics [20], [21], [22]. Such models typically show seasons when mosquitoes are most likely to be active without accounting for climatic factors.

Different modelling frameworks also include the role of migratory birds in WNV transmission. Bergsman et al. [23] formulated an ODE model that investigates the interplay of WNV in both resident and migratory hosts. The model considers the yearly seasonal outbreaks that depend primarily on the number of susceptible migrant birds entering the local population each season, demonstrating that the early growth rates of seasonal outbreaks are more influenced by the migratory than the resident bird population. For Germany, Mbaoma et al. [17] recently developed an ODE model for WNV by incorporating the complete life cycle of *Cx. pipiens* as a vector, and resident birds, migratory birds, and humans as hosts. The study concluded that short-distance migratory birds such as the hooded crow were identified as important hosts in maintaining the enzootic transmission cycle of WNV in Germany. These ecological patterns for bird migration provide the fundamental biological basis for incorporating migratory birds into our model. This is mainly because bird movement patterns may determine where the virus can be introduced and how quickly it can spread during transmission seasons.

Although ODE-based models allow us to understand the transmission dynamics of WNV, they generally do not consider the spatial movement of hosts and vectors across different regions without incorporating additional mechanisims, such as those in multi-patch models or distance-dispersal kernels. For example, Bhowmick et al. [24] used an ODE-based modelling approach in combination with different distance dispersal kernels to describe the spreading process of WNV through migratory birds in Germany. As a promising alternative, researchers have incorporated spatial variation and disease spread across different geographical regions into ODE-based models using partial differential equation (PDE) methods. Lin and Zhu [25] developed a reaction–diffusion PDE model for the spatial spread of WNV in mosquitoes and birds in North America using free-boundary conditions. Another study of WNV propagation was conducted by Maidana and Yang [26], who proposed a spatial model to analyse WNV spread across the USA and calculated the travelling wave speed. The model considers a system of PDEs that describes the movement of mosquito and avian populations through diffusion and advection. Results from this study show that mosquito movements do not play a major role in the spread, while bird advection is an important factor for lower mosquito biting rates.

Maidana and Yang [27], [28] also made significant contributions to the spatial modelling of WNV using PDE models, and their framework is the basis of this study. Unlike previous WNV PDE models in the literature, our model considers not only resident birds but also migratory birds as hosts, thereby capturing seasonal changes in WNV transmission patterns driven by bird migration. Furthermore, we simulate the model in Germany, accounting for spatial heterogeneity, identifying high circulation areas, and giving theoretical insights into the contribution of migratory birds to WNV spread. In Germany, WNV was first detected in August 2018 in birds from the Southeastern region of Berlin [29]. Since then, WNV has been established in Germany and has spread. Germany lies along migration routes of birds between Europe, the Mediterranean, and Africa [30]. A study conducted by Nussbaumer et al. [31] indicates that most migratory birds arrive in the northeastern region of Germany during spring and leave the country in early autumn via the southwestern direction. First WNV strains confirmed in Germany were closely related to strains previously detected in Austria and the Czech Republic [29], indicating a potential route of introduction, and their ability to overwinter in *Cx. pipiens* [32] allows the virus to persist in the long term.

**Fig. 1:**
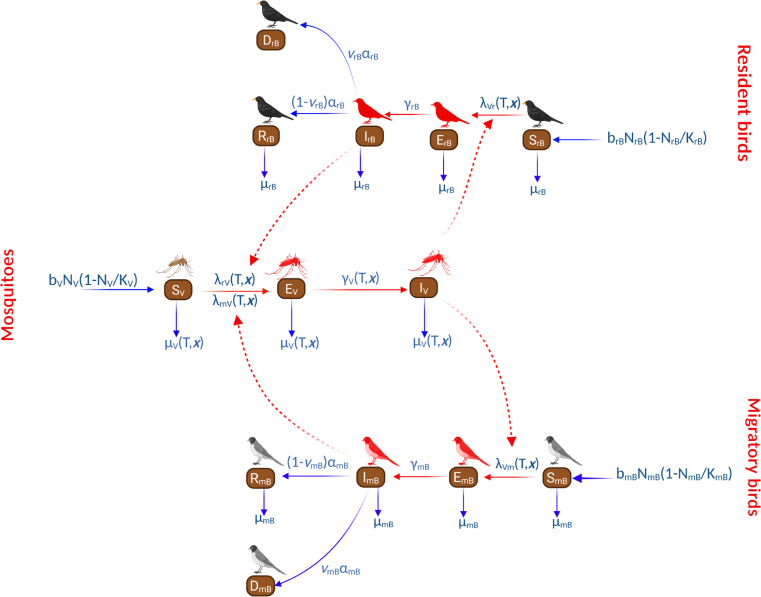
Flow chart diagram describing the transmission dynamics of WNV in Germany. Solid arrows represent transitions between compartments, while dotted lines represent interactions between different states. *Image created in BioRender, with the licence: Arbovirologie (2025)**https://BioRender.com/8a0gfj1**. Content not licensed under the Creative Commons Attribution (CC BY) license.*

## 2 Model formulation

Building on ODE-based models developed by Laperriere et al. [16] and Mbaoma et al. [17], we formulate a model for the spatial spread of WNV in Germany for the years 2019-2025, using 2018 data as initial distribution pattern. Our spatial model consists of a system of semi-linear parabolic PDEs that account for diffusion, advection, and reaction processes, together with temperature-dependent mosquito biting, latency, and mortality rates. In addition, our model includes the populations of mosquitoes and resident and migratory birds. The population of mosquitoes at time t and geographic location x=(x,y), is divided into three compartments: susceptible $S_{V}\left(t,x\right)$, exposed $E_{V}\left(t,x\right)$ and infectious $I_{V}\left(t,x\right)$, with the total population given by $N_{V}\left(t,x\right)=S_{V}\left(t,x\right)+E_{V}\left(t,x\right)+I_{V}\left(t,x\right)$. Mosquitoes are recruited into the susceptible class via a logistic recruitment term: $b_{V}N_{V}\left[1-\frac{N_{V}}{K_{V}}\right]$ governed by the environmental carrying capacity $K_{V}$.

In the susceptible state, mosquitoes get infected by biting an infectious resident bird $I_{rB}$ and/or migratory bird $I_{mB}$ at a temperature-dependent mosquito biting rate

(1) $$\beta \left(T,x\right)=\frac{0.344}{1+1.231e^{-0.184\left(T-20\right)}},$$

and a probability of acquiring the infection of $p_{rV}$ and $p_{mV}$ (Table 1), from resident and migratory birds respectively. When multiple bird host types are present, mosquito bites are distributed among the hosts based on host density and biting preferences. Because of this, we here introduce bite-allocation fractions $q_{r}$ and $q_{m}$, which represent the proportions of mosquito bites on resident and migratory birds, respectively. Under a density-dependent assumption, these fractions are defined as proportional to local host abundance, such that $q_{r}=\frac{N_{rB}}{N_{rB}+cN_{mB}}$ and $q_{m}=\frac{cN_{mB}}{N_{rB}+cN_{mB}}$, where $N_{rB}=S_{rB}+E_{rB}+I_{rB}+R_{rB}$ and $N_{mB}=S_{mB}+E_{mB}+I_{mB}+R_{mB}$ are the total resident and migratory bird populations. c>0 is the host-preference parameter that controls how mosquito bites are divided between resident and migratory birds. When c>1, migratory birds are more attractive, leading to a higher proportion of bites on them. Conversely, when 0<c<1, the attractiveness of migratory birds is reduced, resulting in more bites on resident birds. When c=1, bites are split based on host availability, which corresponds to density-dependent allocation without a specific feeding preference [23].

The force of infection on mosquitoes is therefore given by

$$\lambda_{BV}\left(T,x\right)=\lambda_{r_{V}}+\lambda_{m_{V}}=\frac{q_{r}p_{rV}\beta \left(T,x\right)I_{rB}}{N_{rB}}+\frac{q_{m}p_{mM}\beta \left(T,x\right)I_{mB}}{N_{mB}}.$$

A successful contact between infectious birds and susceptible mosquitoes at location x and time t sends susceptible mosquitoes to the exposed class. The latency rate on mosquitoes was fitted in [15], and is given by

(2) $$\gamma_{V}\left(T,x\right)=\frac{1}{e^{-0.09T+5.36}}.$$

and from the exposed stage, mosquitoes progress to the infectious class $\left(I_{V}\right)$. Mosquitoes die at temperature-dependent natural mortality rate of $\mu_{V}\left(T,x\right)$, where

(3) $$\mu_{V}\left(T,x\right)=\frac{0.0025T^{2}-0.094T+1.0257}{10}$$

defined in [45]. Spatial and temporal plots of the thermal functions (Eqs. (1), (2), and (3)) are presented in Section S1 of the Suplementary material. The host population consists of resident and migratory birds. This population is divided into susceptible $S_{j}$, exposed $E_{j}$, infected $I_{j}$, recovered $R_{j}$, and dead $D_{j}$ hosts, with j=rB,mB, and for easier readability, we denote resident birds by subscript (rB), and migratory birds by (mB). The recruitment into the susceptible bird populations is modelled using a logistic growth with density-dependent recruitment:

$b_{j}N_{j}\left[1-\frac{N_{j}}{K_{j}}\right]$, following Bhowmick et al. [33]. Susceptible resident and migratory birds get infected by interacting with infectious mosquitoes, with a biting rate β(T,x) defined in Eq. (1), and a probability of acquiring the infection of $p_{Vr}$ and $p_{Vm}$ respectively.

In mosquito-borne disease models, the mosquito-to-bird ratio is crucial because it determines the per-bird biting pressure [16], [17], [33], which influences the disease burden. Vogels et al. [34] argued that mosquito abundance is limited by the availability of host blood meals, and bird density directly impacts mosquito feeding success. As a result, the number of bites an individual bird receives mainly depends on the ratio of mosquitoes to birds, rather than solely on mosquito or bird density. Because of this, we introduce the mosquito-to-bird ratio parameter ϕ for resident and migratory birds.

For simplicity of the model, this ratio is considered as an effective seasonal measure of mosquito biting pressure per host, instead of a population count over time. In WNV models, Bhowmick et al. [33] simulated their ODE model with a mosquito-to-bird ratio ranging from 10 to 30, while Laperriere et al. [16] used a ratio of 30. Another study by de Freitas Costa et al. [35], which aimed to investigate the conditions necessary for the circulation of WNV in Germany and the Netherlands, found that larger proportions of susceptible birds and higher vector–host ratios were required. The study showed that WNV circulation in the Netherlands was rarely sustained when vector–host ratios fell below approximately 1:100. We here inferred different yearly ratios whose values ranged between 30 and 60 (Table 2). The force of infection on resident birds is thus given by

(4) $$\lambda_{Vr}\left(T,x\right)=\frac{q_{r}\phi p_{Vr}\beta \left(T,x\right)I_{V}\left(t,x\right)}{N_{V}},$$

while that of migratory birds is

(5) $$\lambda_{Vm}\left(T,x\right)=\frac{q_{m}\phi p_{Vm}\beta \left(T,x\right)I_{V}\left(t,x\right)}{N_{V}}.$$

The latency rates on resident and migratory birds are given by $\gamma_{rB}$ and $\gamma_{mB}$ respectively, while their recovery rates are given by $\alpha_{rB}$ and $\alpha_{mB}$. Proportions $1-\nu_{rB}$ of resident birds and $1-\nu_{mB}$ of migratory birds recover from the infection, while the proportions $\nu_{rB}$ and $\nu_{mB}$ die at the rates $\alpha_{rB}$, and $\alpha_{mB}$ respectively. Resident and migratory bird species can also die naturally at rates $\mu_{rB}$ and $\mu_{mB}$.

### 2.1 Diffusion and advection processes

Diffusion describes the process by which matter is transported from one part of a system to another due to random molecular motions [36]. The concept of diffusion dates back to the 19th century, when Fick [37] recognised the transfer of heat by conduction driven by random molecular motions and quantified this process using the heat-conduction equation previously derived by baron de Fourier [38]. To date, the diffusion process has attracted significant attention, especially in mathematical biology. In ecological modelling, diffusion is used to describe the random movement of species from areas of higher concentration to areas of lower concentration [39], influencing their spatial distribution patterns. On the other hand, the advection process refers to the directional flow of a substance by a known velocity vector field [40]. The combined effects of diffusion and advection, along with the ODE terms, form an advection–diffusion–reaction PDE system that models how a phenomenon spreads over time and space. Reaction terms govern local transmission at each location, while diffusion and advection model the movement of species, including infectious ones.

In this study, we define diffusion for mosquitoes and for both resident and migratory birds as they move locally in search of resting/breeding sites, food, and other needs. The advection process is defined for migratory birds only, as we can account for their seasonal directional migration patterns. Diffusion is denoted by the Laplacian operator (Δ), with diffusion coefficients for mosquitoes, resident, and migratory birds denoted by $D_{1},D_{2},D_{3}$ in ${\text{km}}^{2}/\text{day}$ respectively. Furthermore, we set the diffusion coefficients so that mosquito diffusion is lower than that of resident and migratory birds, as mosquitoes are considered insects with a small radius of action [26], [41], [42]. A lower diffusion coefficient is assigned to resident birds compared to migratory birds to highlight the greater mobility of migratory birds. To improve the interpretability of diffusion coefficients, we define the root-mean-square displacement (RMSD). The RMSD describes the dispersal distance species move from the initial source under diffusion alone after a time τ [43], [44], and it is calculated using the formula:

$$\text{RMSD}=\sqrt{2pD\tau },$$

where p=2 is the spatial dimension, D is the diffusion coefficient and τ is the time step. We denote the advection term by the gradient operator (∇), with a velocity $A=\left[v_{x},v_{y}\right]$ km/day, in a two-dimensional plane. The schematic diagram of our PDE model and the governing system of equations are shown in Fig. 1 and system (6).

(6) Mosquitopopulation:∂SV∂t=D1ΔSV+bVNV1−NVKV−[λrV(T,x)+λmV(T,x)+μV(T,x)]SV,∂EV∂t=D1ΔEV+[λrV(T,x)+λmV(T,x)]SV−γV(T,x)+μV(T,x)EV,∂IV∂t=D1ΔIV+γV(T,x)EV−μV(T,x)IV,

$$\text{Resident birds:}\left\{\begin{array}{cc} \frac{\partial S_{rB}}{\partial t}\; & =\;D_{2}\Delta S_{rB}\;+\;b_{rB}N_{rB}\left[1-\frac{N_{rB}}{K_{rB}}\right]\;-\;\left[\lambda_{Vr}\left(T,x\right)+\mu_{rB}\right]S_{rB},\\ \frac{\partial E_{rB}}{\partial t}\; & =\;D_{2}\Delta E_{rB}\;+\;\lambda_{Vr}\left(T,x\right)S_{rB}\;-\;\left[\gamma_{rB}+\mu_{rB}\right]E_{rB},\\ \frac{\partial I_{rB}}{\partial t}\; & =\;D_{2}\Delta I_{rB}\;+\;\gamma_{rB}E_{rB}\;-\;\left[\alpha_{rB}+\mu_{rB}\right]I_{rB},\\ \frac{\partial R_{rB}}{\partial t}\; & =\;D_{2}\Delta R_{rB}\;+\;\left(1-\nu_{rB}\right)\alpha_{rB}I_{rB}\;-\;\mu_{rB}R_{rB},\\ \frac{\partial D_{rB}}{\partial t}\; & =\;\alpha_{rB}\nu_{rB}I_{rB}, \end{array}\right)$$

$$\text{Migratory birds:}\left\{\begin{array}{cc} \frac{\partial S_{mB}}{\partial t}\; & =\;D_{3}\Delta S_{mB}-A\cdot \nabla S_{mB}\;+\;b_{mB}N_{mB}\left[1-\frac{N_{mB}}{K_{mB}}\right]\;-\;\left[\lambda_{Vm}\left(T,x\right)+\mu_{mB}\right]S_{mB},\\ \frac{\partial E_{mB}}{\partial t}\; & =\;D_{3}\Delta E_{mB}-A\cdot \nabla E_{mB}\;+\;\lambda_{Vm}\left(T,x\right)S_{mB}\;-\;\left[\gamma_{mB}+\mu_{mB}\right]E_{mB},\\ \frac{\partial I_{mB}}{\partial t}\; & =\;D_{3}\Delta I_{mB}-A\cdot \nabla I_{mB}\;+\;\gamma_{mB}E_{mB}\;-\;\left[\alpha_{mB}+\mu_{mB}\right]I_{mB},\\ \frac{\partial R_{mB}}{\partial t}\; & =\;D_{3}\Delta R_{mB}-A\cdot \nabla R_{mB}\;+\;\left(1-\nu_{mB}\right)\alpha_{mB}I_{mB}\;-\;\mu_{mB}R_{mB},\\ \frac{\partial D_{mB}}{\partial t}\; & =\;\nu_{mB}\alpha_{mB}I_{mB}. \end{array}\right)$$

The total populations of mosquitoes, resident birds and migratory birds are given by $N_{V}=S_{V}+E_{V}+I_{V},\;N_{rB}=S_{rB}+E_{rB}+I_{rB}+R_{rB},\;N_{mB}=S_{mB}+E_{mB}+I_{mB}+R_{mB}$, while the initial conditions are given by:

$S_{V}\left(0,x\right)=\psi_{1}\left(x\right)$, $E_{V}\left(0\right)=\psi_{2}\left(x\right)$, $I_{V}\left(0\right)=\psi_{3}\left(x\right)$, $S_{rB}\left(0,x\right)=\psi_{4}\left(x\right)$, $E_{rB}\left(0,x\right)=\psi_{5}\left(x\right)$, $I_{rB}\left(0,x\right)=\psi_{6}\left(x\right)$, $R_{rB}\left(0,x\right)=\psi_{7}\left(x\right)$, $D_{rB}\left(0\right)\geq 0$, $S_{mB}\left(0,x\right)=\psi_{8}\left(x\right)$, $E_{mB}\left(0,x\right)=\psi_{9}\left(x\right)$, $I_{mB}\left(0,x\right)=\psi_{10}\left(x\right)$, $R_{mB}\left(0,x\right)=\psi_{11}\left(x\right)$, $D_{mB}\left(0\right)\geq 0$, where,

$$x=\left(x,y\right)\in \Omega ,\;\Delta =\frac{\partial^{2}\left(\cdot \right)}{\partial x^{2}}+\frac{\partial^{2}\left(\cdot \right)}{\partial y^{2}},\;\nabla =\left[\frac{\partial \left(\cdot \right)}{\partial x},\frac{\partial \left(\cdot \right)}{\partial y}\right].$$

We assume the system moves in a region $\Omega \subset R^{2}$, with a smooth boundary ∂Ω according to Fick’s law [37], so that in the initial conditions, x∈Ω, where $\psi_{i}\in C^{2}\left(\Omega \right)\cap C\left(\overset{¯}{\Omega }\right)$ and subject to the homogeneous Neumann boundary conditions:

$$\frac{\partial S_{V}}{\partial n}=\frac{\partial E_{V}}{\partial n}=\frac{\partial I_{V}}{\partial n}=\frac{\partial S_{rB}}{\partial n}=\frac{\partial E_{rB}}{\partial n}=\frac{\partial I_{rB}}{\partial n}=\frac{\partial R_{rB}}{\partial n}=\frac{\partial S_{mB}}{\partial n}=\frac{\partial E_{mB}}{\partial n}=\frac{\partial I_{mB}}{\partial n}=\frac{\partial R_{mB}}{\partial n}=0,$$

forx∈∂Ω,t>0, and n is the unit outer normal to Ω. The state variables and initial conditions are summarised in Table (S1) of the Supplementary material, while model parameters are shown in Table 1.

**Table 1:** Definition of parameters used in system (6).

Parameter	Definition	Value	Source
$b_{V}\left(x\right)$	Birth rate of mosquitoes	0.0666	[23]
$b_{rB}\left(x\right)$	Birth rate of resident birds	0.00342	[45]
$b_{mB}\left(x\right)$	Birth rate of migratory birds	0.0014	[23]
$\mu_{V}\left(T,x\right)$	Natural mortality rate of mosquitoes	Eq. (3)	[16]
$\mu_{rB}\left(x\right)$	Natural mortality rate of resident birds	0.0005	[17]
$\mu_{mB}\left(x\right)$	Natural mortality rate of migratory birds	0.00023	[17]
$\gamma_{V}\left(T,x\right)$	Latency rate on mosquitoes	Eq. (2)	[15]
$\gamma_{rB}\left(x\right)$	Latency rate on resident birds	0.196	[17]
$\gamma_{mB}\left(x\right)$	Latency rate on migratory birds	0.285	[17]
$\alpha_{rB}\left(x\right)$	Removal rate of infectious resident birds	0.867	[17]
$\alpha_{mB}\left(x\right)$	Removal rate of infectious migratory birds	0.4	[17]
$\nu_{rB}\left(x\right)$	Proportion of dead resident birds	0.655	[17]
$\nu_{mB}\left(x\right)$	Proportion of dead migratory birds	0.103	[17]
$p_{Vr}\left(x\right)$	Probability of a new resident bird infection	0.97	[17]
$p_{Vm}\left(x\right)$	Probability of a new migratory bird infection	0.9	[17]
$p_{rV}\left(x\right)$	Probability of a new mosquito infection from a resident bird	0.4	[17]
$p_{mV}\left(x\right)$	Probability of a new mosquito infection from a migratory bird	0.7	[17]
β(T,x)	Mosquito biting rate	Eq. (1)	[45]
ϕ(x)	Mosquito to resident bird ratio	Table 2	Assumed
$D_{1}\left(x\right)$	Diffusion coefficient of mosquitoes	Table 2	Inferred from the data
$D_{2}\left(x\right)$	Diffusion coefficient of resident birds	Table 2	Inferred from the data
$D_{3}\left(x\right)$	Diffusion coefficient of migratory birds	Table 2	Inferred from the data
$A\left(t,x\right)=\left[v_{x};v_{y}\right]$	Advection vector for migratory birds (in km/day)	[1;1] & [−1;−1]	Estimated Section 5

## 3 Mathematical properties of the model

The mathematical proofs for the positivity, uniqueness, boundedness and well-posedness of the model and its solution are shown in Section S3 of the Supplementary material.

## 4 Steady states and the reproductive number

The PDE system (6) admits a non-trivial WNV-free equilibrium point $E^{0}$ such that

$$\left(S_{V}^{0},E_{V}^{0},I_{V}^{0},S_{rB}^{0},E_{rB}^{0},I_{rB}^{0},R_{rB}^{0},D_{rB}^{0},S_{mB}^{0},E_{mB}^{0},I_{mB}^{0},R_{mB}^{0},D_{mB}^{0}\right)$$

$$=\left(K_{V}\left[1-\frac{\mu_{V}}{b_{V}}\right],0,0,K_{rB}\left[1-\frac{\mu_{rB}}{b_{rB}}\right],0,0,0,0,K_{mB}\left[1-\frac{\mu_{mB}}{b_{mB}}\right],0,0,0,0\right).$$

$E^{0}$ is positive if and only if $b_{j}>\mu_{j}$, for j=V,rB,mB. The uniqueness of $E^{0}$ can be verified using [[46], Lemma 2.1]. The basic reproductive number of the deterministic part of our model is computed following [47], as follows:

Let ℱ, be the matrix of new infections, and V, the matrix of transitions, then

$$ℱ=\left[\begin{array}{c} \left(\lambda_{rV}+\lambda_{Vm}\right)S_{V}\\ 0\\ \lambda_{Vr}S_{rB}\\ 0\\ \lambda_{Vm}S_{mB}\\ 0 \end{array}\right],\;V=\left[\begin{array}{c} \left(\gamma_{V}+\mu_{V}\right)E_{V}\\ -\gamma_{V}E_{V}+\mu_{V}I_{V}\\ \left(\gamma_{rB}+\mu_{rB}\right)E_{rB}\\ -\gamma_{rB}E_{rB}+\left(\alpha_{rB}+\mu_{rB}\right)I_{rB}\\ \left(\gamma_{mB}+\mu_{mB}\right)E_{mB}\\ -\gamma_{mB}E_{mB}+\left(\alpha_{mB}+\mu_{mB}\right)I_{mB} \end{array}\right].$$

Partial derivatives evaluated at the WNV-free equilibrium point yield:

$$F=\left[\begin{array}{cccccc} 0 & 0 & 0 & \frac{\phi \beta p_{rV}S_{V}^{0}}{S_{rB}^{0}+S_{mB}^{0}} & 0 & \frac{\phi \beta p_{mV}S_{V}^{0}}{S_{rB}^{0}+S_{mB}^{0}}\\ 0 & 0 & 0 & 0 & 0 & 0\\ 0 & \frac{\beta p_{Vr}S_{rB}^{0}}{S_{V}^{0}\left(S_{rB}^{0}+S_{mB}^{0}\right)} & 0 & 0 & 0 & 0\\ 0 & 0 & 0 & 0 & 0 & 0\\ 0 & \frac{\beta p_{Vm}S_{mB}^{0}}{S_{V}^{0}\left(S_{rB}^{0}+S_{mB}^{0}\right)} & 0 & 0 & 0 & 0\\ 0 & 0 & 0 & 0 & 0 & 0 \end{array}\right],$$

and

$$V=\left[\begin{array}{cccccc} -\gamma_{V}-\mu_{V} & 0 & 0 & 0 & 0 & 0\\ \gamma_{V} & -\mu_{V} & 0 & 0 & 0 & 0\\ 0 & 0 & -\gamma_{rB}-\mu_{rB} & 0 & 0 & 0\\ 0 & 0 & \gamma_{rB} & -\alpha_{rB}-\mu_{rB} & 0 & 0\\ 0 & 0 & 0 & 0 & -\gamma_{mB}-\mu_{mB} & 0\\ 0 & 0 & 0 & 0 & \gamma_{mB} & -\alpha_{mB}-\mu_{mB} \end{array}\right].$$

The basic reproductive number $R_{0}\left(x\right)$ is thus given by the spectral radius of $FV^{-1}$, which gives

(7) $$R_{0}\left(x\right)=\sqrt{R_{0}^{Res}+R_{0}^{Mig}},$$

$$\text{where}\;R_{0}^{Res}=\frac{\gamma_{V}\phi \beta p_{rV}p_{Vr}\gamma_{rB}}{\mu_{V}\left(\alpha_{rB}+\mu_{rB}\right)\left(\gamma_{rB}+\mu_{rB}\right)\left(\gamma_{V}+\mu_{V}\right){\left[1+\frac{K_{mB}\left(1-\frac{\mu_{mB}}{b_{mB}}\right)}{K_{rB}\left(1-\frac{\mu_{rB}}{b_{rB}}\right)}\right]}^{2}}$$

$$\text{and}\;R_{0}^{Mig}=\frac{\gamma_{V}\phi \beta p_{mV}p_{Vm}\gamma_{mB}}{\mu_{V}\left(\alpha_{mB}+\mu_{mB}\right)\left(\gamma_{mB}+\mu_{mB}\right)\left(\gamma_{V}+\mu_{V}\right){\left[1+\frac{K_{rB}\left(1-\frac{\mu_{rB}}{b_{rB}}\right)}{K_{mB}\left(1-\frac{\mu_{mB}}{b_{mB}}\right)}\right]}^{2}}.$$

**Fig. 2:**
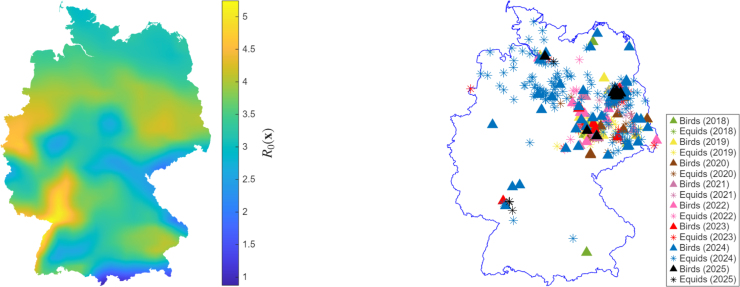
The basic reproductive number $R_{0}\left(x\right)$ compared to observed data.

Since all parameters of the model are positive, and the equilibrium point is also positive, then $R_{0}^{Res}$ and $R_{0}^{Mig}$ are non-negative. The basic reproductive number $R_{0}\left(x\right)$ is plotted for the entire country and compared with observed data for 2018–2025 (Fig. 2). $R_{0}\left(x\right)$ shows high circulation areas in the eastern parts of Germany, with other regions in the southeastern and northern parts also showing circulation, which is in agreement with the observed data. Moreover, we also included the 2025 case data to evaluate whether our $R_{0}\left(x\right)$ risk map can predict regions where future cases may occur. Indeed, as of March 2026, 2025 WNV cases were observed in the east, north, and southwest of Germany, regions previously flagged as high-risk by $R_{0}\left(x\right)$. This result is important because it allows potential high-risk regions to be identified before seasonal outbreaks occur.

## 5 Simulation framework

Based on available WNV data in Germany, birds of the Accipitridae family were the most affected species in 2018 [17], [48]. Therefore, as also done by Mbaoma et al. [17], we used parameters associated with northern goshawks and raptors for the resident bird, both of which fall under the Accipitridae family. Migratory birds in the model represent species that move over short to long distances, particularly along the northeast to southwest seasonal migratory routes. Parameters for these birds are based on those observed in corvids, as they are among the most frequently reported WNV-positive birds, second only to raptors and tits [17]. For a detailed derivation of these parameters, we refer the reader to [17] and the references therein.

Initial population densities of infectious birds and mosquitoes are estimated to be high in Halle (Saale), the main WNV hot spot in 2018, followed by Berlin, and, lastly, lower densities in Laage, Poing, and Plessa, as indicated by the 2018 WNV observed case data [49]. On the other hand, initial densities of susceptible hosts and mosquitoes are assumed to be spatially homogeneously distributed across the entire country. A 2-dimensional Gaussian function of the form

$$f\left(t,x\right)=e^{-\frac{{\left(x-x_{0}\right)}^{2}+{\left(y-y_{0}\right)}^{2}}{2\sigma^{2}}}$$

is used to define the initial condition peaks. $\left(x_{0},y_{0}\right)$ are the central coordinates specifying the nodes of the infected regions, and σ is the standard deviation. σ=40 km in Berlin and Halle Saale (main hotspot regions), and 20 km in other infected areas.

The effect of host-specific feeding allocation is first examined using a baseline scenario with c=1, in which mosquito feeding preference depends solely on bird densities. This scenario is used to infer diffusion coefficients and compare model predictions with observed data. Two additional scenarios are then defined: scenario 1, with c=0.4, in which mosquitoes primarily feed on resident birds; and scenario 2, with c=3, in which mosquitoes prefer migratory birds.

The model runs on a daily time step from 1 March to 30 November each year, spanning 2019 to 2025. Each year, the final solution for that year is saved and used as the initial condition for the next, but starting with zero exposed and infectious birds. Such an initialisation reflects the short duration of WNV viremia in birds, which lasts only a few days [50], [51], [52], and may be cleared before the next spring. The only reservoirs considered in this work are mosquitoes, which overwinter with infected specimens carrying the virus into spring [32], [53]. These start and end dates are chosen because WNV activity is known to be high in summer. Spring and autumn are included to account for the seasonal bird migrations into Germany in spring and out in autumn. This choice also enables the incorporation of new data from ongoing WNV surveillance activities. For instance, the initial conditions for 2020 were updated when a new WNV case was confirmed in Hamburg, north of Germany, which may have influenced subsequent spread patterns observed in the north.

In the process, we use inverse modelling to infer the diffusion and advection coefficients that may have influenced the observed spreading pattern. This is done by running the model with different coefficients and visually comparing the model output to the observed data. The final coefficients are selected based on their ability to replicate the observed pattern, while respecting the biological constraints of the magnitudes, i.e., $D_{1}<D_{2}<D_{3}$. This approach is primarily used because the available data are limited and sparse; therefore, our main goal is to explain the observed qualitative spread pattern of WNV.

Temperature strongly influences the activity of *Cx. pipiens* including their ability to transmit WNV [54]. This motivated us to model mosquito parameters using temperature-dependent functions, namely: mosquito biting rate β(T,x), latency rate $\gamma_{V}\left(T,x\right)$, and mortality rate $\mu_{V}\left(T,x\right)$. ERA5 reanalysis data on single levels from 1940 to present are downloaded from the Copernicus Climate Data Store [55]. The data is first cleaned, and the years 2019–2025 are subsequently extracted for interpolation onto the mesh and used to compute the temperature-space dependent mosquito functions. During the simulation process, $\beta \left(T,x\right),\gamma_{V}\left(T,x\right)$ and μ(T,x) are dynamically updated based on the temperatures at different locations in Germany. Advection, representing the directed movement of migratory birds, is fixed throughout the years. The spring-to-early summer movement is described by the vector field $\left[v_{x};v_{y}\right]=\left[1;1\right]$ while the late summer-to-autumn movement is described by $\left[v_{x};v_{y}\right]=\left[-1;-1\right]$ in km per day. To solve our PDE model, we use the MATLAB PDE Toolbox [56], which uses the finite element method. The toolbox solves PDEs of the form:

(8) $$m\frac{\partial^{2}u}{\partial t^{2}}+d\frac{\partial u}{\partial t}-\nabla \cdot \left(c\nabla u\right)+au=f,$$

In our case, m=0, d=1, and a=0. Matrices c and f represent the diffusion coefficients and reaction terms, respectively, and are included in Section S4 of the Supplementary material, along with the pseudo-code for the simulation procedure. The map of Germany is downloaded as a shapefile from [57] and simplified using MapShaper [58]. The observed data indicate presence only, as no absence data are available, and for this reason, we qualitatively present our results on a Low-High scale for better interpretation.

## 6 Model fitting

To fit our model predictions, we compared simulated cases with observed data. The observed data comprises bird and equid infection cases, as well as mortality, obtained from the World Organisation for Animal Health (WOAH) [59]. Due to the lack of systematic WNV surveillance in Germany, many dead birds go undetected, while others are discovered at decomposed stages, which can affect PCR tests. Domesticated equids are usually easier to diagnose because owners often report sick animals. Given that birds are the amplifying hosts, it is reasonable to assume that, before equid cases are reported, an amplifying host was infected first and might have gone undetected. Moreover, our observed bird data is dominated by dead birds, while the equid data is dominated by infected equids. Because of this, our model fitting compares merged WNV-related events observed in birds and equids, with the compartment of dead resident birds, given the high WNV-related deaths observed in birds of the Accipitridae family [17], [48]. Firstly, we aggregate both the simulated and observed data within the GADM (Database of Global Administrative Areas) level-2 administrative units of Germany (N = 356) [57], comprising both rural districts (Landkreise) and urban districts (kreisfreie Städte). The PDE solution $u\left(x,y,t_{f}\right)$ is extracted at the final time step, and the domain Ω is divided into 356 sub domains ${\left\{\Omega_{i}\right\}}_{i=1}^{N}$ corresponding to the GADM level-2 administrative units, such that $\Omega ={⋃}_{i=1}^{N}\Omega_{i}$. For each $\Omega_{i}$, the mean simulated number of cases is given by $\frac{1}{\left|\Omega_{i}\right|}\int_{\Omega_{i}}u\left(x,y,t_{f}\right)\;dA$, where $\left|\Omega_{i}\right|$ is the area of the region $\Omega_{i}$.

Observed cases are aggregated using the formula $\sum_{j=1}^{N_{\text{obs}}}1_{\Omega_{i}}\left(x_{j},y_{j},t_{f}\right)$, where $1_{\Omega_{i}}\left(x_{j},y_{j},t_{f}\right)$ is the indicator function equal to 1 if the observed point lies within $\Omega_{i}$, and 0 otherwise, and $N_{\text{obs}}$ is the number of observed points per region.

Boundaries are shrunk inwards using the polybuffer function [56] from the toolbox to prevent misclassification near borders. The isinterior operator [56] is then applied to prevent double-counting elements located on regional borders. Thus, only points lying strictly within the interior of a polygon are counted as belonging to that region.

Lastly, Spearman’s rank correlation coefficient (ρ) [60] is applied to quantify the degree of spatial correspondence between simulated and observed patterns in each district. Spearman’s (ρ) is calculated using the formula:

(9) $$\rho =1-\frac{6\overset{N}{\underset{i=1}{\sum }}d_{i}^{2}}{N\left(N^{2}-1\right)},$$

where $d_{i}$ is the difference between the rank of the simulated and observed points, and N is the number of administrative units. A positive ρ indicates that locations with a high density of simulated cases correspond to locations with a high density of observed points. Conversely, a negative ρ implies that high values in one pattern correspond to low values in the other, indicating an opposite relationship. A ρ close to zero suggests the absence of a monotonic spatial relationship [60]. P-values are computed to assess whether the observed correlation occurred by chance, with smaller p-values (< 0.05) indicating that the correlation is unlikely to have occurred by chance.

## 7 Results

**Fig. 3:**
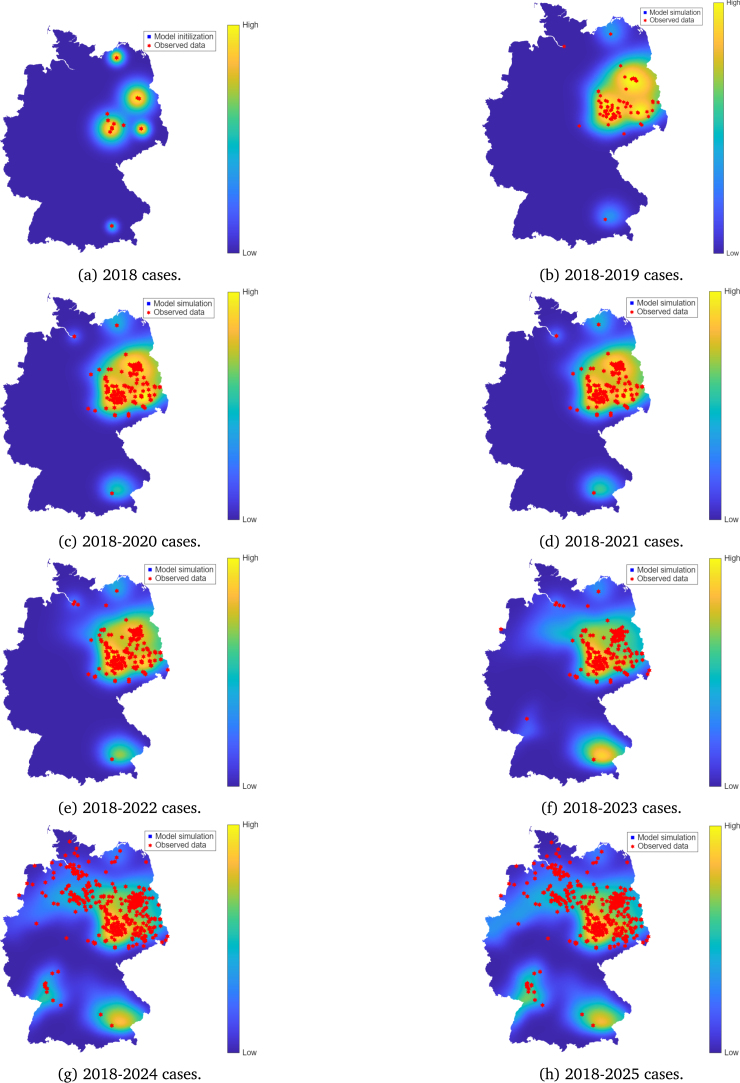
Model simulation compared to observed data of WNV in Germany between the years 2019–2025, for the case when c=1, corresponding to density-dependent bite allocation proportional to host availability.

**Fig. 4:**
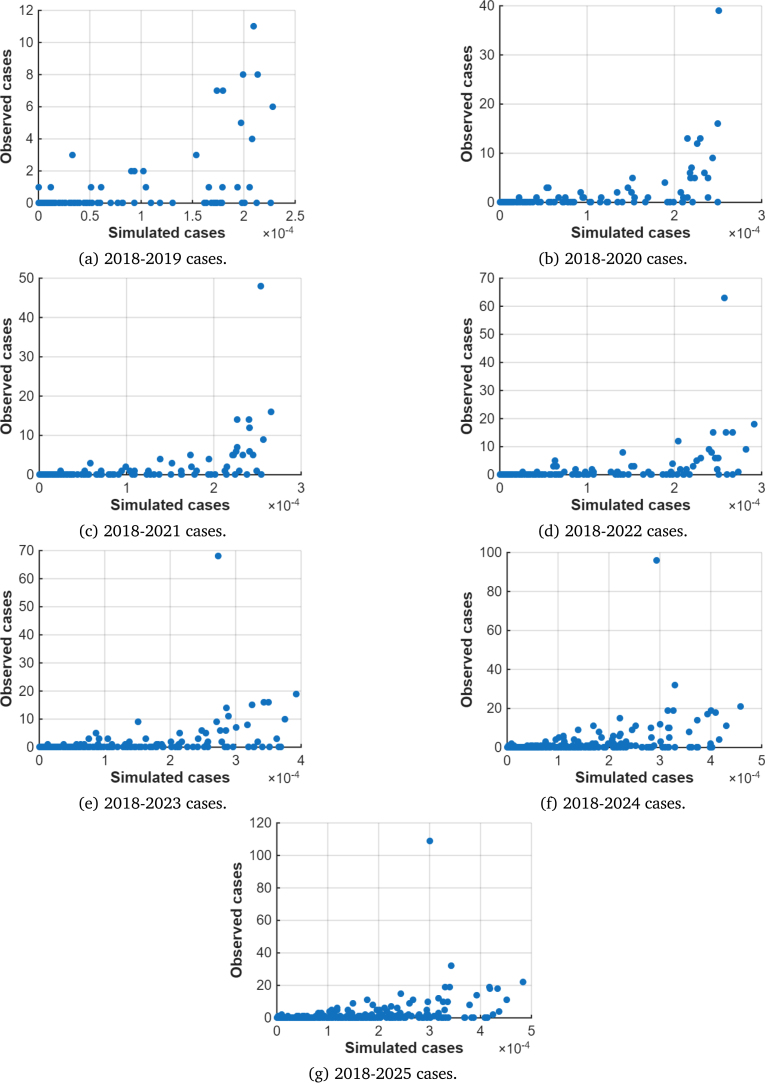
Scatter plots for Spearman’s rank correlation showing the monotonic relationship between model predictions and observed data for the case c=1, corresponding to density-dependent bite allocation proportional to host availability.

For all scenarios, the model is initialised with 2018 WNV cases data. Under the baseline scenario, where mosquito biting is solely influenced by host availability, a strong visual agreement between the simulated and observed patterns is evident throughout the 2019–2025 simulation period (Fig. 3). The model’s main strength lies in its ability to identify high circulation areas that were initially localised in 2018. The RMSD estimates indicate that WNV-mosquito infections reached an average annual dispersal range of 46.9 km in 2022, and 33.2 km for all other years. However, resident birds dispersed an average range of 66.3 km in 2022 and 57.4 km in all other years. In migratory birds, an annual RMSD of 104.9 km was observed over the years (Table 2). The RMSD for mosquitoes remained lower than that for resident and migratory birds, while the RMSD for resident birds was lower than that for migratory birds, reflecting the greater mobility of avian hosts compared to mosquito species. Still under the baseline scenario, Spearman’s correlation coefficients (ρ) were all statistically significant with values greater than 0.4 (Table 2, Fig. 4).

In 2023, the model accurately predicted an isolated case in the northwestern region (next to the Dutch border) and another isolated case in the southwestern region close to the Upper Rhine valley (Fig. 3(f)). This suggests the model’s ability to anticipate under-reported spread patterns mainly driven by temperature, mosquito, and bird movements.

When mosquito preference is biased towards resident birds (scenario 1, c=0.4), the model predicts a slower spatial expansion of WNV (Fig. 7). Although resident birds may experience increased local transmission, the reduced infection probability among migratory birds limits long-range dissemination of the virus. Only in 2025 did the model manage to detect the isolated case observed in 2023 along the Upper Rhine valley. This outcome suggests that local amplification alone is insufficient to reproduce the observed national-scale spread and highlights the critical role of migratory-mediated dispersal, which can involve either short or long-distance migration, in shaping WNV spreading patterns.

In contrast, when mosquitoes bite migratory birds instead of resident birds (scenario 3, c=3), the spread of WNV becomes rapid (Fig. 6). This occurs when mosquito bites that might have gone to resident birds are instead taken by migratory birds. It is clear that by the year 2022, cases observed in 2024 were already being predicted by the model. Due to their directional dispersal patterns, infected migratory birds could serve as long-range carriers, establishing the virus in new locations beyond primary endemic areas. Therefore, host-specific feeding behaviour might play a crucial role in long-distance transmission dynamics.

**Fig. 5:**
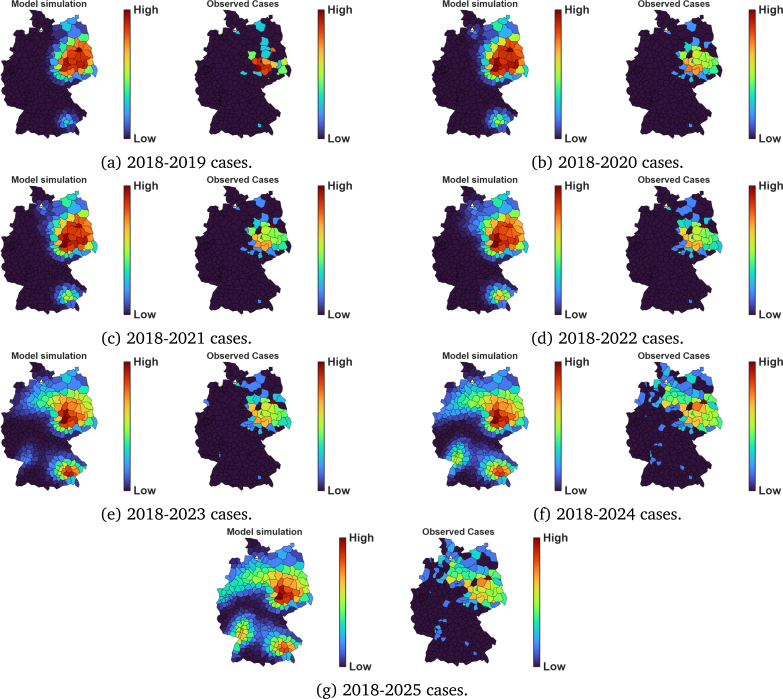
Spatial comparison between simulated and observed WNV cases in Germany (2019–2025), on a $\log_{10}$ scale.

**Table 2:** Summary values for the diffusion coefficients $\left(D_{1},D_{2},D_{3}\right),$ in km${}^{2}$ per day; RMSD for each species in km; Spearman’s correlation coefficient and P-values, for the case c=1, corresponding to density-dependent bite allocation proportional to host availability.

Year	D${}_{1}$	D${}_{2}$	D${}_{3}$	RMSD${}_{1}$	RMSD${}_{2}$	RMSD${}_{3}$	$\phi_{V}$	ρ	P-value
2019	1.0	3.0	10.0	33.2	57.4	104.9	60	0.42	$5.9\times 10^{-16}$
2020	1.0	3.0	10.0	33.2	57.4	104.9	30	0.49	$2.3\times 10^{-25}$
2021	1.0	3.0	10.0	33.2	57.4	104.9	40	0.48	$3.4\times 10^{-23}$
2022	2.0	4.0	10.0	46.9	66.3	104.9	50	0.50	$1.3\times 10^{-22}$
2023	1.0	3.0	10.0	33.2	57.4	104.9	50	0.44	$1.0\times 10^{-17}$
2024	1.0	3.0	10.0	33.2	57.4	104.9	30	0.42	$1.1\times 10^{-16}$
2025	1.0	3.0	10.0	33.2	57.4	104.9	30	0.43	$4.9\times 10^{-17}$

**Fig. 6:**
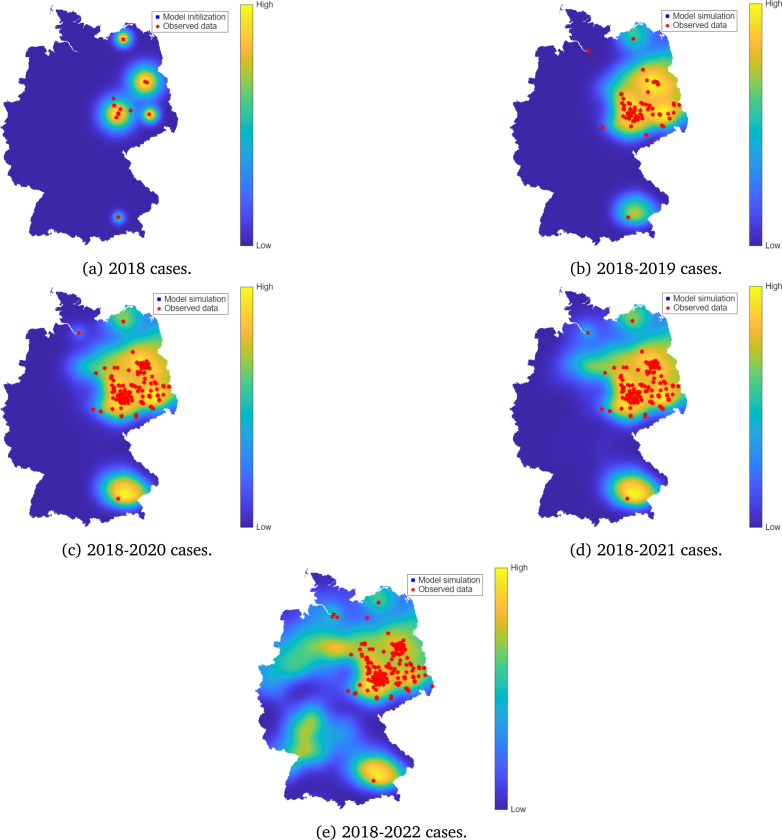
Model simulations compared with observed WNV cases in Germany (2019–2022) under Scenario 3: c=3, representing preferential biting of migratory birds.

**Fig. 7:**
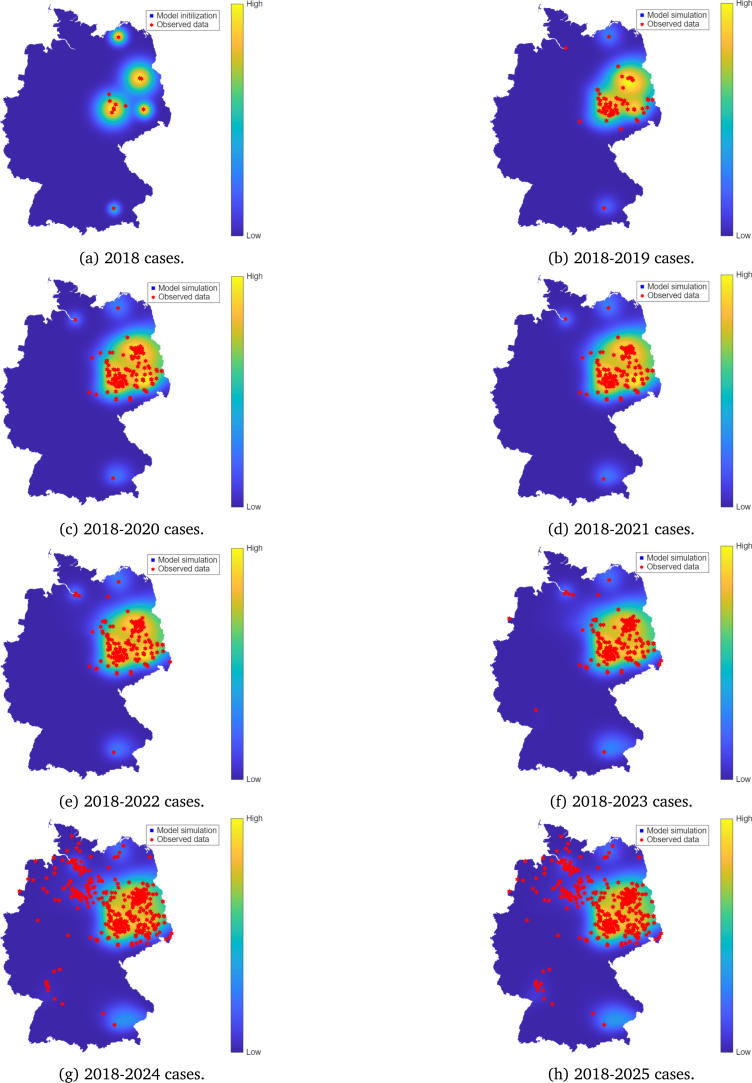
Model simulation compared to observed data of WNV in Germany between the years 2019–2025, for the case when c=0.4, corresponding to the case when resident birds are preferred, and there is a reduced mosquito feeding on migratory birds.

## 8 Discussions and conclusions

Understanding the WNV spreading pattern allows early detection of WNV outbreaks. Identifying high-circulation areas also supports early planning and resource allocation for preventive measures. At the same time, implementing systematic surveillance programs to understand the spread of WNV is costly. Therefore, in this study, we modelled the spread of WNV in Germany using a temperature-driven PDE model with advection and diffusion processes. We proved that our model has non-negative, bounded, and unique solutions, and that the system (6) is well-posed.

In agreement with previous studies [17], [23], [33], at the temporal level, the basic reproductive number $\left(R_{0}\left(x\right)\right)$ indicates that the presence of migratory birds increases the number of amplifying local hosts. At the spatial scale, $R_{0}\left(x\right)$ identified low-high risk regions of public health interest, which agreed with the observed WNV cases in birds and equids, and other temperature-driven WNV models, e.g., by Mbaoma et al. [17]. The reproductive number identified regions in the eastern parts of Germany as high circulation areas, a result similar to that obtained in [17], [24]. In addition, $R_{0}\left(x\right)$ flagged regions in the north, south and southwest of Germany as new circulation areas. Our approach explains the drivers of WNV spread in Germany and infers estimates of spreading speeds. In particular, from the simulation of our PDE model, we computed the annual RMSD, which estimates the average displacement of the infection over a given period, and due to a lack of real host movement data, we chose diffusion coefficients that best matched the observed data.

Scenario analyses comparing the model with different mosquito biting preferences between resident and migratory birds were explored. Results indicated that when a large proportion of bites is allocated to resident birds, WNV spread is very localised (Fig. 7). In contrast, when migratory birds receive more bites, a more rapid spread is observed (Fig. 6) and the model detected 2023, 2024, and 2025 cases as early as in 2022 (Fig. 6(e)). While this comparison is based on limited, sparse data, the alignment between simulated spreading pathways and known migratory bird flyways is consistent with the hypothesis that long-range, directed bird migration may contribute to WNV dissemination. This observation is also supported by a recent study by Tóth et al. [61]. Our results suggest that migratory birds may still have played a role in shaping the WNV spread pattern in Germany, even if they may not be the primary source of infection. Thus, effective surveillance and prevention efforts of WNV may also be implemented at key stopover sites, where migratory birds are likely to rest and interact with local mosquitoes.

The 2023 data showed an isolated WNV case near the Dutch border (Fig. 3(f)). That year, no WNV cases were reported directly across the Dutch border, suggesting that the German case may have resulted from internal dispersal rather than immediate cross-border importation. However, given regional differences in surveillance intensity and reporting, the absence of reported cases may not necessarily indicate the absence of infection. Therefore, while the spatial agreement is promising, results near national boundaries should be interpreted with caution, given our modelling assumptions on homogeneous Neumann boundary conditions, which prevent cross-border inflows. Notably, in 2023, the model identified a region between the Dutch border and parts of the north of Germany as a possible high-circulation area, a finding confirmed by the observational data for the year 2024 (Fig. 2 and Fig. 3(f)).

Our model uncovers a transmission corridor for WNV that spreads from the east in an anticlockwise direction, likely reflecting the temperature gradient suitable for mosquito activity. Interestingly, the circulation areas identified in our model agree with those in the study by Di Pol et al. [62], which aimed at modelling the temperature suitability for the risk of WNV establishment in European *Cx. pipiens* populations. As of March 2026, WNV bird and equid cases for 2025 had been reported in the eastern, northern, and southwestern regions, regions that were previously flagged as circulation areas in previous years (Fig. 2, right). This is an indication of seasonal recurrence supported by empirical evidence from North America and Southern Europe WNV events that show that once introduced into ecologically suitable regions, WNV tends to establish persistent seasonal transmission with annual reemergence [7], [63]. The overwintering of WNV-infected *Culex* mosquitoes likely plays an important role for the local persistence of the virus [32].

Results from our spatially explicit PDE model suggest that temperature, mosquito-feeding preference, together with species movement through diffusion and advection, play a significant role in the spread of WNV in Germany. However, while our model successfully simulates WNV dispersal in Germany, limitations exist. For instance, factors such as land use influence the overall spatiotemporal distribution of mosquito populations [64], which is important for modelling mosquito abundance. Additionally, we used only temperature as the weather variable to account for heterogeneity in mosquito parameters, whereas precipitation, wind speed, and wind direction also affect mosquito distribution and activity [65]. If spatially resolved datasets for all these factors are accessible, they can be considered to improve the spatial heterogeneity of our PDE model. Estimating diffusion and advection coefficients through inverse modelling may introduce uncertainty and potential identifiability issues, as different parameter combinations can yield similar model outputs [66], [67]. Even if our diffusion coefficients were estimated within a biologically plausible range consistent with previous studies and known dispersal behaviour of species, exploratory simulations (not presented here) indicated that the qualitative model behaviour remained reasonable even for different combinations, and thus values chosen here represent one plausible scenario. Future work could address this limitation by adopting more systematic estimation frameworks, such as Bayesian approaches [68]. To better capture spatial heterogeneity, another step could be to use spatially varying diffusion coefficients, since movement patterns are not constant in reality. Moreover, our PDE model could also be extended to a stochastic PDE system to incorporate additional random effects that deterministic PDEs cannot model.

The low correlation coefficients observed in model fitting may have partly resulted from the sparse and discrete nature of the reported observational data. Future work incorporating higher-resolution surveillance data could improve the model’s calibration accuracy. Furthermore, the choropleth maps presented in Fig. 5 indicate that the model overestimates cases in some towns, where no WNV cases were confirmed. Although this could be true given the continuous nature of the PDE model, the lack of systematic surveillance might also lead to under-reporting of observed cases. This is evidenced by the fact that most data arises from incidental or passive detection rather than systematic monitoring [69], and thus our simulation results should be viewed as minimum estimates rather than representing the full WNV situation.

## CRediT authorship contribution statement

**Pride Duve:** Writing – review & editing, Conceptualization, Methodology, Formal analysis, Software, Validation, Writing – original draft. **Felix Gregor Sauer:** Writing – review & editing, Writing – original draft, Supervision, Conceptualization. **Renke Lühken:** Writing – review & editing, Writing – original draft, Supervision, Conceptualization.

## Declaration of competing interest

The authors declare that they have no known competing financial interests or personal relationships that could have influenced the work reported in this paper.

## Data Availability

The MATLAB codes used to reproduce the simulations presented in this study, together with the cleaned WNV datasets used for model fitting, are archived at Zenodo and can be accessed from Duve et al. [70]. The codes may also be adapted to investigate transmission dynamics in other regions. The original datasets can be obtained from the World Organisation for Animal Health (WOAH) [59] website.

## Appendix A Supplementary data

Video S7

MMC S8 Supplementary material containing mathematical analysis of the model and figures for temperature-dependent functions. MMC S8
