# Supplementary material for: Modelling the impact of temperature and bird migration on the spread of West Nile virus
Source: One Health. 2026 Mar 11;22:101386. doi: 10.1016/j.onehlt.2026.101386 (PMC12999322; doi:10.1016/j.onehlt.2026.101386)
Supplement: MMC S8 — Supplementary material containing mathematical analysis of the model and figures for temperature-dependent functions. [file mmc8.pdf]

Supplementary File: Modelling the impact of temperature and bird migration on the spread of West Nile virus

Pride Duve*, Felix Gregor Sauer, and Renke Lühken

Bernhard Nocht Institute for Tropical Medicine, Hamburg, Germany

S1 Plots for mosquito parameters

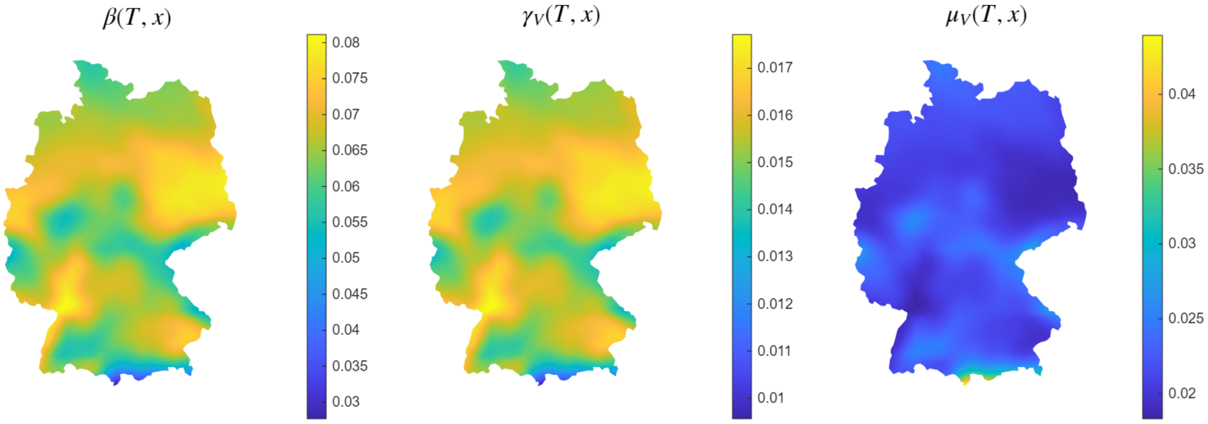

(a) Space-dependent thermal mosquito parameters for the year 2024.

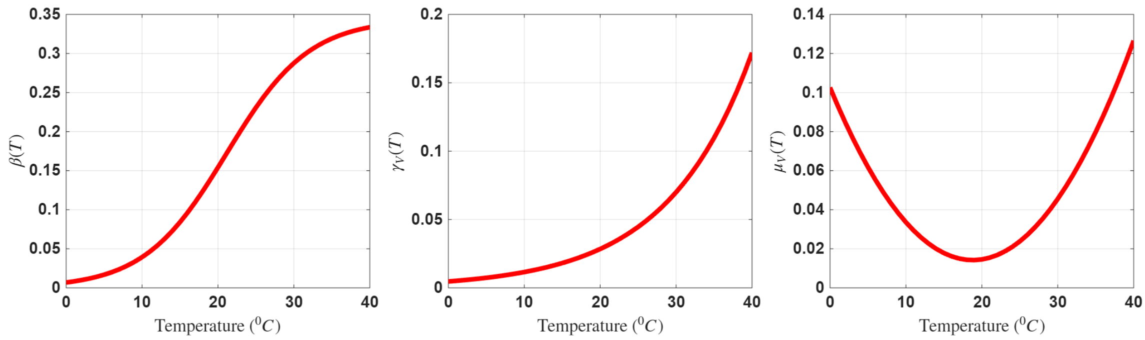

(b) Temperature evolution for mosquito parameters.

Figure S1: Spatio-temporal mosquito parameters used in the model. The parameters $\beta(T,x)$ and $\mu_V(T,x)$ are adapted from [1], while $\gamma_V(T,x)$ from [2].

S2 Initial conditions

Unlike ODE-based models that rely on total population counts, spatially explicit PDE models interpret state variables as population densities, typically measured in individuals per unit area. Thus, it is important to convert the ODE-based initial conditions in Table (S1) into population densities per

*Corresponding author: `pride.duve@bnitm.de`

unit area by dividing susceptible classes by the total area of our mesh (cleaned map of Germany). From the mesh, we obtained a total domain area of approximately A = 356,280 km^2 for mainland Germany. The other compartments are localized around the infection points for 2018, and thus they are only divided by the total area around the infection, which we denote B.

Table S1: Definition of variables used in system (6), as defined by the authors.

Variable	Definition	Value	Units
$S_V(0,\boldsymbol{x})$	susceptible mosquitoes	$20 \times 10^9/$A	mosquitoes/m^2
$E_V(0,\boldsymbol{x})$	exposed mosquitoes	$10,000/$B	mosquitoes/m^2
$I_V(0,\boldsymbol{x})$	infectious mosquitoes	$10,000/$B	mosquitoes/m^2
$S_{rB}(0,\boldsymbol{x})$	susceptible resident birds	$110 \times 10^6/$A	birds/m^2
$E_{rB}(0,\boldsymbol{x})$	exposed resident birds	$10,000/$B	birds/m^2
$I_{rB}(0,\boldsymbol{x})$	infectious resident birds	$10,000/$B	birds/m^2
$R_{rB}(0,\boldsymbol{x})$	recovered resident birds	$10,000/$B	birds/m^2
$D_{rB}(0,\boldsymbol{x})$	dead resident birds	$10,000/$B	birds/m^2
$S_{mB}(0,\boldsymbol{x})$	susceptible migratory birds	$5 \times 10^5/$A	birds/m^2
$E_{mB}(0,\boldsymbol{x})$	exposed migratory birds	$10,000/$B	birds/m^2
$I_{mB}(0,\boldsymbol{x})$	infectious migratory birds	$10,000/$B	birds/m^2
$R_{mB}(0,\boldsymbol{x})$	recovered migratory birds	$10,000/$B	birds/m^2
$D_{mB}(0,\boldsymbol{x})$	dead migratory birds	$10,000/$B	birds/m^2

S3 Mathematical properties of the model

Theorem 1. *System (6) has non-negative and unique solutions that are bounded in* $[0,\infty)$.

Proof. We write system (6) together with the corresponding initial conditions, in the Banach space of continuous functions that include the boundary, $\mathscr{B} = C(\bar{\Omega})$, as follows [3]:

$$\begin{cases} \dfrac{\partial X(t,\boldsymbol{x})}{\partial t} = & \mathscr{L}X(t,\boldsymbol{x}) + f(X(t,\boldsymbol{x})),\ t>0, \\ X(t,0) = & X_0 \geq 0_{\mathbb{R}^{11}}, \end{cases} \tag{S1}$$

where

$$X(t,\boldsymbol{x})=\begin{bmatrix} S_V(t,\boldsymbol{x})\\ E_V(t,\boldsymbol{x})\\ I_V(t,\boldsymbol{x})\\ S_{rB}(t,\boldsymbol{x})\\ E_{rB}(t,\boldsymbol{x})\\ I_{rB}(t,\boldsymbol{x})\\ R_{rB}(t,\boldsymbol{x})\\ S_{mB}(t,\boldsymbol{x})\\ E_{mB}(t,\boldsymbol{x})\\ I_{mB}(t,\boldsymbol{x})\\ R_{mB}(t,\boldsymbol{x}) \end{bmatrix},\ X(0,\boldsymbol{x})=\begin{bmatrix} S_V(0,\boldsymbol{x})\\ E_V(0,\boldsymbol{x})\\ I_V(0,\boldsymbol{x})\\ S_{rB}(0,\boldsymbol{x})\\ E_{rB}(0,\boldsymbol{x})\\ I_{rB}(0,\boldsymbol{x})\\ R_{rB}(0,\boldsymbol{x})\\ S_{mB}(0,\boldsymbol{x})\\ E_{mB}(0,\boldsymbol{x})\\ I_{mB}(0,\boldsymbol{x})\\ R_{mB}(0,\boldsymbol{x}) \end{bmatrix},\ \mathscr{L}X(t,\boldsymbol{x})=\begin{bmatrix} D_1\Delta S_V(t,\boldsymbol{x})\\ D_1\Delta E_V(t,\boldsymbol{x})\\ D_1\Delta I_V(t,\boldsymbol{x})\\ D_2\Delta S_{rB}(t,\boldsymbol{x})\\ D_2\Delta E_{rB}(t,\boldsymbol{x})\\ D_2\Delta I_{rB}(t,\boldsymbol{x})\\ D_2\Delta R_{rB}(t,\boldsymbol{x})\\ D_3\Delta S_{mB}(t,\boldsymbol{x})-\boldsymbol{A}\cdot\nabla S_{mB}(t,\boldsymbol{x})\\ D_3\Delta E_{mB}(t,\boldsymbol{x})-\boldsymbol{A}\cdot\nabla E_{mB}(t,\boldsymbol{x})\\ D_3\Delta I_{mB}(t,\boldsymbol{x})-\boldsymbol{A}\cdot\nabla I_{mB}(t,\boldsymbol{x})\\ D_3\Delta R_{mB}(t,\boldsymbol{x})-\boldsymbol{A}\cdot\nabla R_{mB}(t,\boldsymbol{x}) \end{bmatrix},$$

and

$$f=\begin{cases} f_1 = & b_V N_V\left[1-\dfrac{N_V}{K_V}\right]-[\lambda_{rV}(T)+\lambda mV(T)+\mu_V]S_V,\\ f_2 = & [\lambda_{rV}(T)+\lambda mV(T)]S_V-(\gamma_V+\mu_V)E_V,\\ f_3 = & \gamma_V E_V-\mu_V I_V,\\ f_4 = & b_{rB}N_{rB}\left[1-\dfrac{N_{rB}}{K_{rB}}\right]-[\lambda_{Vr}(T)+\mu_{rB}]S_{rB},\\ f_5 = & \lambda_{Vr}(T)S_{rB}-[\gamma_{rB}+\mu_{rB}]E_{rB},\\ f_6 = & \gamma_{rB}E_{rB}-[\alpha_{rB}+\mu_{rB}]I_{rB},\\ f_7 = & (1-\nu_{rB})\alpha_{rB}I_{rB}-\mu_{rB}R_{rB},\\ f_8 = & b_{mB}N_{mB}\left[1-\dfrac{N_{mB}}{K_{mB}}\right]-[\lambda_{Vm}(T)+\mu_{mB}]S_{mB},\\ f_9 = & \lambda_{Vm}(T)S_{mB}-[\gamma_{mB}+\mu_{mB}]E_{mB},\\ f_{10} = & \gamma_{mB}E_{mB}-[\alpha_{mB}+\mu_{mB}]I_{mB},\\ f_{11} = & (1-\nu_{mB})\alpha_{mB}I_{mB}-\mu_{mB}R_{mB}. \end{cases}$$

Next, we show that f is locally Lipschitz in $\mathscr{B}$. Thus we show that

$$\forall\, K\subset\mathscr{B},\ \exists L:\quad \|f(X_1)-f(X_2)\|_\infty\le L\|X_1-X_2\|_\infty,\ \forall X_1,X_2\in K,$$

where L is the Lipschitz constant.

We observe that for $\Lambda_V=b_V N_V\left[1-\dfrac{N_V}{K_V}\right]$, $\Lambda_{rB}=b_{rB}N_{rB}\left[1-\dfrac{N_{rB}}{K_{rB}}\right]$ and $\Lambda_{mB}=b_{mB}N_{mB}\left[1-\dfrac{N_{mB}}{K_{mB}}\right]$,

$$X_1 - X_2 = \begin{bmatrix} [\Lambda_{V_1} - \Lambda_{V_2}] - [\lambda_{rV_1} S_{V_1} - \lambda_{rV_2} S_{V_2}] - [\lambda_{mV_1} S_{V_1} - \lambda_{mV_2} S_{V_2}] - \mu_V (S_{V_1} - S_{V_2}) \\ [\lambda_{rV_1} S_{V_1} - \lambda_{rV_2} S_{V_2}] + [\lambda_{mV_1} S_{V_1} - \lambda_{mV_2} S_{V_2}] - (\gamma_V + \mu_V)[E_{V_1} - E_{V_2}] \\ \gamma_V [E_{V_1} - E_{V_2}] - \mu_V [I_{V_1} - I_{V_2}] \\ [\Lambda_{rB_1} - \Lambda_{rB_2}] - (\lambda_{Vr_1} S_{rB_1} - \lambda_{Vr_2} S_{rB_2}) - \mu_{rB}[S_{rB_1} - S_{rB_2}] \\ \lambda_{Vr_1} S_{rB_1} - \lambda_{Vr_2} S_{rB_2} - (\gamma_{rB} + \mu_{rB})[E_{rB_1} - E_{rB_2}] \\ \gamma_{rB}[E_{rB_1} - E_{rB_2}] - (\alpha_{rB} + \mu_{rB})[I_{rB_1} - I_{rB_2}] \\ (1 - \nu_{rB})\alpha_{rB}[I_{rB_1} - I_{rB_2}] - \mu_{rB}[R_{rB_1} - R_{rB_2}] \\ [\Lambda_{mB_1} - \Lambda_{mB_2}] - [\lambda_{Vm_1} S_{mB_1} - \lambda_{Vm_2} S_{mB_2}] - \mu_{mB}[S_{mB_1} - S_{mB_2}] \\ \lambda_{Vm_1} S_{mB_1} - \lambda_{Vm_2} S_{mB_2} - (\gamma_{mB} + \mu_{mB})[E_{mB_1} - E_{mB_2}] \\ \gamma_{mB}[E_{mB_1} - E_{mB_2}] - (\alpha_{mB} + \mu_{mB})[I_{mB_1} - I_{mB_2}] \\ (1 - \nu_{mB})\alpha_{mB}[I_{mB_1} - I_{mB_2}] - \mu_{mB}[R_{mB_1} - R_{mB_2}] \end{bmatrix}.$$

Simplifying, we obtain $\|f(X_1) - f(X_2)\|_\infty =$

$$\begin{aligned}
&= \sup_{x\in\bar\Omega} |[\Lambda_{V_1} - \Lambda_{V_2}] - S_{V_1}[\lambda_{rV_1} - \lambda_{rV_2}]| \\
&\vee \sup_{x\in\bar\Omega} |-\lambda_{rV_2}[S_{V_1} - S_{V_2}] - S_{V_1}[\lambda_{mV_1} - \lambda_{mV_2}] - \lambda_{mV_2}[S_{V_1} - S_{V_2}] - \mu_V(S_{V_1} - S_{V_2})| \\
&\vee \sup_{x\in\bar\Omega} |\gamma_V[E_{V_1} - E_{V_2}] - \mu_V[I_{V_1} - I_{V_2}]| \\
&\vee \sup_{x\in\bar\Omega} |[\Lambda_{rB_1} - \Lambda_{rB_2}] - \lambda_{Vr_1}[S_{rB_1} - S_{rB_2}] - S_{rB_2}[\lambda_{Vr_1} - \lambda_{Vr_2}] - \mu_{rB}[S_{rB_1} - S_{rB_2}]| \\
&\vee \sup_{x\in\bar\Omega} |\lambda_{Vr_1}[S_{rB_1} - S_{rB_2}] + S_{rB_2}[\lambda_{Vr_1} - \lambda_{Vr_2}] - (\gamma_{rB} + \mu_{rB})[E_{rB_1} - E_{rB_2}]| \\
&\vee \sup_{x\in\bar\Omega} |\gamma_{rB}[E_{rB_1} - E_{rB_2}] - (\alpha_{rB} + \mu_{rB})[I_{rB_1} - I_{rB_2}]| \\
&\vee \sup_{x\in\bar\Omega} |(1 - \nu_{rB})\alpha_{rB}[I_{rB_1} - I_{rB_2}] - \mu_{rB}[R_{rB_1} - R_{rB_2}]| \\
&\vee \sup_{x\in\bar\Omega} |[\Lambda_{mB_1} - \Lambda_{mB_2}] - \lambda_{Vm_1}[S_{mB_1} - S_{mB_2}] - S_{mB_2}[\lambda_{Vm_1} - \lambda_{Vm_2}] - \mu_{mB}[S_{mB_1} - S_{mB_2}]| \\
&\vee \sup_{x\in\bar\Omega} |\lambda_{Vm_1}[S_{mB_1} - S_{mB_2}] + S_{mB_2}[\lambda_{Vm_1} - \lambda_{Vm_2}] - (\gamma_{mB} + \mu_{mB})[E_{mB_1} - E_{mB_2}]| \\
&\vee \sup_{x\in\bar\Omega} |\gamma_{mB}[E_{mB_1} - E_{mB_2}] - (\alpha_{mB} + \mu_{mB})[I_{mB_1} - I_{mB_2}]| \\
&\vee \sup_{x\in\bar\Omega} |(1 - \nu_{mB})\alpha_{mB}[I_{mB_1} - I_{mB_2}] - \mu_{mB}[R_{mB_1} - R_{mB_2}]|.
\end{aligned} \tag{S2}$$

By the triangular inequality, and some simplifications, we arrive at

$$\|f(X_1) - f(X_2)\|_\infty \le (\mu_V + \nu_{rB}\alpha_{rB} + \nu_{mB}\alpha_{mB}) \|X_1 - X_2\|_\infty,$$

and thus f is locally Lipschitz in $\mathscr{B}$. By [[4], Theorem B.17], [5], and [[6], Theorem 14.4], there exist a local smooth and unique solution of system (6) in Ω. We observe that system (6) can be written in the form of system (14.12) in the book [6], together with initial data defined in system (14.13). By [[6], Theorem (14.14)], the solutions of system (6) are always positive. □

S3.1 Well-posedness of the model in a feasible region

Theorem 2. *System (6) is well-posed mathematically and biologically, and for any* $(S_V(0,x), E_V(0,x), I_V(0,x), S_{rB}(0,x), E_{rB}(0,x), I_{rB}(0,x), R_{rB}(0,x), S_{mB}(0,x), E_{mB}(0,x), I_{mB}(0,x), R_{mB}(0,x)) \in \mathbb{X}$, *system (6) admits a unique positive solution:* $(S_V(t,x), E_V(t,x), I_V(t,x), S_{rB}(t,x), E_{rB}(t,x), I_{rB}(t,x), R_{rB}(t,x), S_{mB}(t,x), E_{mB}(t,x), I_{mB}(t,x), R_{mB}(t,x)) \in \mathbb{X}$, *satisfying:* $(S_V(t,x), E_V(t,x), I_V(t,x), S_{rB}(t,x), E_{rB}(t,x), I_{rB}(t,x), R_{rB}(t,x), S_{mB}(t,x), E_{mB}(t,x), I_{mB}(t,x), R_{mB}(t,x) \in C^{1,2}((0,\infty)\times\bar{\Omega}) \times C^{1,2}((0,\infty)\times\bar{\Omega})$, *where* $\mathbb{X} := C(\bar{\Omega}) \times C(\bar{\Omega})$.

Moreover, there exist another constant $C_1 > 0$ *independent of initial data, such that the solution* $(S_V(t,x), E_V(t,x), I_V(t,x), S_{rB}(t,x), E_{rB}(t,x), I_{rB}(t,x), R_{rB}(t,x), S_{mB}(t,x), E_{mB}(t,x), I_{mB}(t,x), R_{mB}(t,x))$ *satisfies:*

$\|S_V(t,x)\|_{L^\infty(\Omega)} + \|E_V(t,x)\|_{L^\infty(\Omega)} + \|I_V(t,x)\|_{L^\infty(\Omega)} + \|S_{rB}(t,x)\|_{L^\infty(\Omega)} + \|E_{rB}(t,x)\|_{L^\infty(\Omega)} + \|I_{rB}(t,x)\|_{L^\infty(\Omega)} + \|R_{rB}(t,x)\|_{L^\infty(\Omega)} + \|S_{mB}(t,x)\|_{L^\infty(\Omega)} + \|E_{mB}(t,x)\|_{L^\infty(\Omega)} + \|I_{mB}(t,x)\|_{L^\infty(\Omega)} + \|R_{mB}(t,x)\|_{L^\infty(\Omega)} \le C_1$, *for all* $t > T_0 > 0$.

Proof. We follow the approach presented in [7, 8]. Using the regularity theory of parabolic PDEs [9], system (6) admits a unique non-negative classical solution

$$(S_j(t,x), E_j(t,x), I_j(t,x), R_j(t,x)) \in C^{1,2}((0,T_m)\times\bar{\Omega}) \times C^{1,2}((0,T_m)\times\bar{\Omega}),$$

where T_m represents the maximal existence time of the solution. By the strong maximum principle [10], then $S_j(t,x), E_j(t,x), I_j(t,x), R_j(t,x)$, where $j = rB, mB$ are positive in $(0,T_m)\times\bar{\Omega}$.

Summing up the equations gives

$$\frac{\partial N_j}{\partial t} = D_j \Delta N_j + b_j N_j \left[1 - \frac{N_j}{K_j}\right] - \mu_j N_j - \alpha_j \nu_j I_j,$$

subject to homogeneous Neumann boundary conditions $\nabla N_j \cdot n = 0$ on $\partial\Omega$ and non-negative initial data $N_j(x,0) = N_j(x) \ge 0$. Integrating over Ω, we get

$$\frac{d}{dt}\int_\Omega N_j(x,t)\,dx = \int_\Omega [b_j - \mu_j] N_j\,dx - \int_\Omega b_j \frac{N_j}{K_j}\,dx \le \bar{r}\int_\Omega N_j(x,t)\,dx,\ x \in \Omega,\ t > 0, \bar{r} \le b_{j,max},$$

yielding

$$\int_\Omega N_j(x,t)\,dx \le \int_\Omega e^{t b_{j,max}} N_{j0}(x)\,dx, \quad x \in \Omega,\ t \ge 0.$$

Thus, $\|S_j(t,\cdot)\|_{L^1(\Omega)}$, $\|E_j(t,\cdot)\|_{L^1(\Omega)}$, $\|I_j(t,\cdot)\|_{L^1(\Omega)}$, and $\|R_j(t,\cdot)\|_{L^1(\Omega)}$ are bounded for all $0 < t < T_m$. By the positivity of $S_j(t,\cdot)$, $E_j(t,\cdot)$, $I_j(t,\cdot)$, $R_j(t,\cdot)$, and [[7], Lemma (3.1)], with $\sigma = p_0 = 1$, we conclude that there exist a positive constant C_1 that does not depend on initial data such that the solution S_j, E_j, I_j, R_j satisfies

$$\|S_j(t,\cdot)\|_{L^\infty(\Omega)} + \|E_j(t,\cdot)\|_{L^\infty(\Omega)} + \|I_j(t,\cdot)\|_{L^\infty(\Omega)} + \|R_j(t,\cdot)\|_{L^\infty(\Omega)} \le C_1,\ \forall t > T_0.$$

Similar arguments can be made for the mosquito population, thus we conclude that system (6) is well-posed. □

S4 Simulation framework

The toolbox solves PDEs of the form

$$m\frac{\partial^2 u}{\partial t^2} + d\frac{\partial u}{\partial t} - \nabla \cdot (c\nabla u) + au = f, \tag{S3}$$

and in our case, $m = 0, d = 1$, $a = 0$, and matrices c and f are given by:

$$c = [D_1; D_1; D_1; D_2; D_2; D_2; D_2; 0; D_3; D_3; D_3; D_3; 0],$$

while

$$f = \begin{cases} \mu_V(T,\boldsymbol{x})N_V \quad - \quad [\lambda_{rV}(T,\boldsymbol{x}) + \lambda mV(T,\boldsymbol{x}) + \mu_V(T,\boldsymbol{x})]S_V, \\ [\lambda_{rV}(T,\boldsymbol{x}) + \lambda mV(T,\boldsymbol{x})]S_V \quad - \quad [\gamma_V(T,\boldsymbol{x}) + \mu_V(T,\boldsymbol{x})]\,E_V, \\ \gamma_V(T,\boldsymbol{x})E_V \quad - \quad \mu_V(T,\boldsymbol{x})I_V, \\ \mu_{rB}N_{rB} \quad - \quad [\lambda_{Vr}(T,\boldsymbol{x}) + \mu_{rB}]\,S_{rB}, \\ \lambda_{Vr}(T,\boldsymbol{x})S_{rB} \quad - \quad [\gamma_{rB} + \mu_{rB}]E_{rB}, \\ \gamma_{rB}E_{rB} \quad - \quad [\alpha_{rB} + \mu_{rB}]I_{rB}, \\ (1-\nu_{rB})\alpha_{rB}I_{rB} \quad - \quad \mu_{rB}R_{rB}, \\ \alpha_{rB}\,\nu_{rB}I_{rB}, \\ \mu_{mB}N_{mB} \quad - \quad [\lambda_{Vm}(T,\boldsymbol{x}) + \mu_{mB}]\,S_{mB} - \boldsymbol{A}\cdot\nabla S_{mB}, \\ \lambda_{Vm}(T,\boldsymbol{x})S_{mB} \quad - \quad [\gamma_{mB} + \mu_{mB}]E_{mB} - \boldsymbol{A}\cdot\nabla E_{mB}, \\ \gamma_{mB}E_{mB} \quad - \quad [\alpha_{mB} + \mu_{mB}]I_{mB} - \boldsymbol{A}\cdot\nabla I_{mB}, \\ (1-\nu_{mB})\alpha_{mB}I_{mB} \quad - \quad \mu_{mB}R_{mB} - \boldsymbol{A}\cdot\nabla R_{mB}, \\ \nu_{mB}\alpha_{mB}I_{mB}. \end{cases} \tag{S4}$$

The matrix c consists of the diffusion coefficients, while f stores reaction and advection terms. The model is solved as follows [11]:

Algorithm S1 Pseudo-code for solving system (7) using Matlab's `PDEToolbox`

Step 1: Create the PDE model with N equations

`model` ← `createpde(N)`

Step 2: Define the geometry

`geometryFromEdges(model,g)`

Step 3: Generate a mesh for the geometry

`gm` ← `generateMesh(model)`

Step 4: Add boundary conditions to the PDEModel

`applyBoundaryCondition(_)`

Step 5: Specify coefficients of the PDE model

`specifyCoefficients(model,"m",0,"d",1,"c",c,"a",0,"f",f)`

Step 6: Give initial conditions or initial solution

`setInitialConditions(model,initial_value_vector)`

Step 7: Solve the PDE system

`results` ← `solvepde(model)`

Step 8: Extract the solution

`Sol` ← `results.NodalSolution`

Step 9: Plot the solution

Plot `Sol` using `pdeplot()`

The map of Germany is downloaded as a shape file from (https://gadm.org/download_country_v3.html) and simplified using mapshaper [12]. Matlab codes for the full implementation and the data are freely available in our Zenodo repository [13] and they can be used to extend our model, or adopt for any country.

References

[1] Vincent Laperriere, Katharina Brugger, and Franz Rubel. Simulation of the seasonal cycles of bird, equine and human west nile virus cases. *Preventive Veterinary Medicine*, 98(2–3):99–110, February 2011. ISSN 0167-5877. https://dx.doi.org/10.1016/j.prevetmed.2010.10.013.

[2] Julian Heidecke, Jonas Wallin, Peter Fransson, Pratik Singh, Henrik Sjödin, Pascale Claire Stiles, Marina Treskova, and Joacim Rocklöv. Uncovering temperature sensitivity of west nile virus transmission: Novel computational approaches to mosquito-pathogen trait responses. *PLOS Computational Biology*, 21(3):e1012866, March 2025. ISSN 1553-7358. doi: 10.1371/journal.pcbi.1012866. URL http://dx.doi.org/10.1371/journal.pcbi.1012866.

[3] Hamadjam Abboubakar, Reinhard Racke, and Nicolas Schlosser. A reaction-diffusion model for the transmission dynamics of the coronavirus pandemic with reinfection and vaccination process. 2023.

[4] Vincenzo Capasso. *Mathematical Structures of Epidemic Systems*. Springer Berlin Heidelberg, 1993. ISBN 9783540705147. https://dx.doi.org/10.1007/978-3-540-70514-7.

[5] Xavier Mora. Semilinear parabolic problems define semiflows on c k spaces. *Transactions of the American Mathematical Society*, 278(1):21, July 1983. ISSN 0002-9947. https://dx.doi.org/10.2307/1999300.

[6] Joel Smoller. *Shock Waves and Reaction—Diffusion Equations*. Springer US, 1983. ISBN 9781468401523. https://dx.doi.org/10.1007/978-1-4684-0152-3.

[7] Rui Peng and Xiao-Qiang Zhao. A reaction–diffusion sis epidemic model in a time-periodic environment. *Nonlinearity*, 25(5):1451–1471, April 2012. ISSN 1361-6544. https://dx.doi.org/10.1088/0951-7715/25/5/1451.

[8] Jianpeng Wang and Binxiang Dai. Qualitative analysis on a reaction-diffusion host-pathogen model with incubation period and nonlinear incidence rate. *Journal of Mathematical Analysis and Applications*, 514(2):126322, October 2022. ISSN 0022-247X. https://dx.doi.org/10.1016/j.jmaa.2022.126322.

[9] C. V. Pao. *Nonlinear parabolic and elliptic equations*. Springer US, 1993. ISBN 9781461530343. https://dx.doi.org/10.1007/978-1-4615-3034-3.

[10] Murray H. Protter and Hans F. Weinberger. *Maximum principles in differential equations*. Springer New York, 1984. ISBN 9781461252825. https://dx.doi.org/10.1007/978-1-4612-5282-5.

[11] The MathWorks, Inc. *MATLAB*. Natick, Massachusetts, United States, 2024. Available from https://www.mathworks.com/help/pde/pde-problem-setup.html?s_tid=CRUX_topnav.

[12] M. Harrower and M. Bloch. Mapshaper.org: a map generalization web service. *IEEE Computer Graphics and Applications*, 26(4):22–27, July 2006. ISSN 0272-1716. doi: 10.1109/mcg.2006.85. URL http://dx.doi.org/10.1109/mcg.2006.85.

[13] Pride Duve, Felix. G. Sauer, and Renke Lühken. Codes and data for the paper: Modelling the impact of temperature and bird migration on the spread of West Nile Virus, June 2025. URL https://doi.org/10.5281/zenodo.18996831. Version 1.0.
